# Hypothetical Roles of the Olfactory Tubercle in Odor-Guided Eating Behavior

**DOI:** 10.3389/fncir.2020.577880

**Published:** 2020-11-11

**Authors:** Koshi Murata

**Affiliations:** ^1^Division of Brain Structure and Function, Faculty of Medical Sciences, University of Fukui, Fukui, Japan; ^2^Life Science Innovation Center, Faculty of Medical Science, University of Fukui, Fukui, Japan

**Keywords:** olfaction, olfactory tubercle, eating, dopamine, motivation, palatability, attractive behavior, aversive behavior

## Abstract

Olfaction plays an important role in the evaluation, motivation, and palatability of food. The chemical identity of odorants is coded by a spatial combination of activated glomeruli in the olfactory bulb, which is referred to as the odor map. However, the functional roles of the olfactory cortex, a collective region that receives axonal projections from the olfactory bulb, and higher olfactory centers in odor-guided eating behaviors are yet to be elucidated. The olfactory tubercle (OT) is a component of the ventral striatum and forms a node within the mesolimbic dopaminergic pathway. Recent studies have revealed the anatomical domain structures of the OT and their functions in distinct odor-guided motivated behaviors. Another component of the ventral striatum, the nucleus accumbens, is well known for its involvement in motivation and hedonic responses for foods, which raises the possibility of functional similarities between the OT and nucleus accumbens in eating. This review first summarizes recent findings on the domain- and neuronal subtype-specific roles of the OT in odor-guided motivated behaviors and then proposes a model for the regulation of eating behaviors by the OT.

## Introduction

The smell of food stimulates appetite, especially during states of hunger. Conversely, the smell of rotten foods incites a sense of discomfort and promotes arousal. The sense of olfaction is also involved in mastication. Volatile flavor compounds move through the nasopharynx and reach the olfactory mucosa, a process termed as retronasal olfaction. Therefore, the sense of “taste” and pleasure of palatable tastes is attenuated if the nose is pinched during chewing or drinking. Gustatory, tactile, and olfactory inputs of foods are integrated during “tasting” and create a sense of “flavor,” subsequently resulting in the palatability of foods. Thus, olfaction is involved in the evaluation, appetite, and palatability of food before and during eating (Lawless, 1991; Shepherd, 2013).

Odorants are volatile chemical molecules that can be detected by olfactory sensory neurons in the olfactory epithelium. Each olfactory sensory neuron expresses a single type of odorant receptor that has a particular molecular receptive range and sends axons to a specific glomerulus in the olfactory bulb, the first relay center of the central olfactory system (Mori and Sakano, 2011). Odorants are coded by specific combinations of activated glomeruli, termed “odor maps,” in the olfactory bulb (Uchida et al., 2000). The brain regions that receive synaptic inputs from projection neurons in the olfactory bulb (mitral and tufted cells) are collectively referred to as the olfactory cortex (Neville and Haberly, 2004). The olfactory cortex includes the anterior olfactory nucleus, tenia tecta, piriform cortex, olfactory tubercle (OT), cortical amygdala, and entorhinal cortex. In contrast to that in the olfactory bulb, the spatial combination of activated neurons in the piriform cortex does not seem to represent the chemical identities of odorants (Stettler and Axel, 2009). Axonal projections from the olfactory bulb to the olfactory cortex are diffused and dispersed (Ghosh et al., 2011; Sosulski et al., 2011). To date, the nature of the information encoded by neural activity in the olfactory cortex has not been elucidated. Furthermore, the neural mechanisms underpinning odor-guided evaluation, appetite, and palatability of foods remain unclear.

The OT is a component of the olfactory cortex based on the definition that it receives direct inputs from the olfactory bulb and has been observed in every mammal studied including humans and rodents to date. The term “olfactory tubercle” is used to designate the region on the basal surface of the frontal lobe between the olfactory tract and the nucleus of the diagonal band in humans (Crosby and Humphrey, 1941; Allison, 1954). The OT also forms the ventral striatum, is anatomically bridged to the nucleus accumbens (NAc), and is a component of the mesolimbic dopaminergic pathway (Heimer, 1978; Heimer et al., 1987; Ikemoto, 2010). The OT can efficiently induce intracranial self-administration of addictive drugs (Ikemoto, 2003; Ikemoto et al., 2005; Shin et al., 2008), which are hallmarks of reward processing in the brain (Berridge and Kringelbach, 2015). Recent studies have revealed the involvement of the OT in motivated behaviors, including eating. In this review, I first outline how anatomical maps of the OT match the functional domains of distinct motivated behaviors. I then propose hypothetical roles of the OT in eating behaviors based on the sense of olfaction.

### Functional Domains of the Olfactory Tubercle and Odor-Guided Motivated Behaviors

**FIGURE 1:**
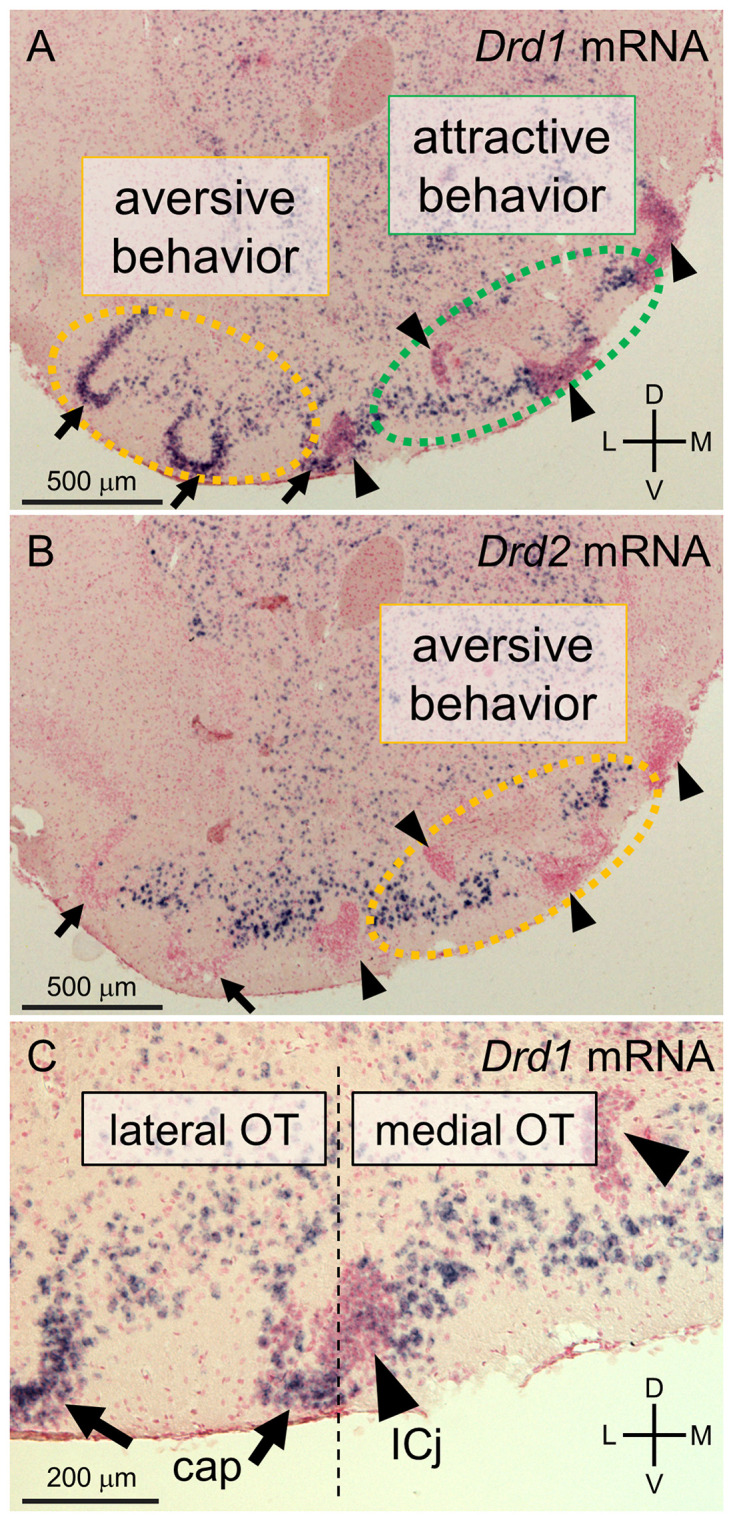
Cytoarchitectonically defined-domains of the mouse olfactory tubercle (OT). **(A–C)** *In situ* hybridization for dopamine receptor D1 (*Drd1*, **A,C**) and D2 (*Drd2*, **B**) mRNA in the anterior OT of mouse counterstained with Nuclear Fast Red. *c-fos* expression mapping revealed that D1 receptor-expressing neurons in the anteromedial OT were activated by sugar-associated cue odors, which accompanied attractive behavior **(A)** and that D1 receptor-expressing neurons in the lateral OT **(A)** and D2 receptor-expressing neurons in the anteromedial OT **(B)** were activated by electrical shock-associated cue odors which accompanied aversive behavior. In the cap regions (arrows), the dwarf cells are densely packed and express *Drd1* but not *Drd2* mRNA **(B,C)**. The granule cells in the Islands of Calleja (arrowheads) are also densely packed but exhibit weak expression of D*rd1* and no *Drd2* mRNA **(B,C)**. Medium spiny neurons are mainly distributed in the layer II of the cortex-like regions, which are interposed between the cap regions and Islands of Calleja in these coronal sections, and express either *Drd1* or *Drd2* **(A,B)**. The cap region and Islands of Calleja serve as anatomical landmarks for the lateral and medial domains of the OT, respectively. ICj, Islands of Calleja; D, dorsal; V, ventral; M, medial; L, lateral. The Figures are modified from (Murata et al., 2015).

The principal neurons in most areas of the olfactory cortex are pyramidal-type glutamatergic neurons. By contrast, the majority of neurons in the OT, as a component of the striatum, are small to medium-sized spiny GABAergic neurons (Millhouse and Heimer, 1984; Neville and Haberly, 2004). The cytoarchitecture of the OT comprises three major neuronal types: medium spiny neurons distributed in layer II of the cortex-like region; dwarf cells, which are small spiny neurons constituting the cap regions (Hosoya and Hirata, 1974); and granule cells, which are also small GABAergic neurons constituting the Islands of Calleja (Fallon et al., 1978; de Vente et al., 2001). These structural divisions can be observed based on the mRNA expression levels of the dopamine receptors D1 and D2 (Figure 1; Murata et al., 2015). Medium spiny neurons in the cortex-like region express either *Drd1* or *Drd2* mRNA (Figures 1A,B). The dwarf cells in the cap region express *Drd1* but not *Drd2* mRNA (Figures 1B,C). The granule cells in the Islands of Calleja are characterized by weak expression of *Drd1* mRNA and the absence of *Drd2* mRNA (Figures 1B,C). The cap region and Islands of Calleja are also distinguishable by the expression of DARPP-32, as the cap regions are immunopositive and the Islands of Calleja are immunonegative for DARPP-32, respectively (Ouimet et al., 1984; Murata et al., 2015). The cap regions run in an anteroposterior direction through the lateral part of the OT. In contrast, the Islands of Calleja run in an anteroposterior direction through the anteromedial superficial layer to the posteromedial deep layer (Murata et al., 2015; Xiong and Wesson, 2016).

A recent study revealed that spatially segregated domains of the OT are involved in distinct motivated behaviors. Learned odor-induced attractive and aversive behaviors accompanied *c-fos* expression in distinct domains of the OT (Murata et al., 2015). When mice exhibited attractive behaviors to a cue odor paired with the presence of sugar, *c-fos* expression increased in D1 receptor-expressing medium spiny neurons in the anteromedial domain. Conversely, when mice exhibited aversive behaviors to a cue odor paired with an electrical foot shock, *c-fos* expression was increased in D1 receptor-expressing medium spiny neurons and dwarf cells in the lateral domain and D2 receptor-expressing medium spiny neurons in the anteromedial domain. These findings suggest that the spatial pattern of neural activation in the OT encodes odor-induced attractive or aversive behaviors, in contrast to the odor map of the olfactory bulb. Consistent with this, electrophysiological recordings in behaving mice revealed that the firing activity of the OT flexibly encoded the valence of conditioned odors over odorant identity (Gadziola et al., 2015, 2020). These studies suggest that OT neurons evaluate odorants in an experience-dependent manner and that spatially segregated domains and neuronal subtypes play distinct roles in inducing appropriate motivated behaviors.

The involvement of the medial domain of the OT in attractive behaviors has been demonstrated in several reports. The anteromedial OT was the most effective region that elicited intracranial self-administration of cocaine among a range of distinct striatal regions including the anteromedial OT, anterolateral OT, posteromedial OT, NAc shell and core, and dorsal striatum (Ikemoto, 2003). Silencing of the medial OT in estrous female mice with the inhibitory designer receptor exclusively activated by designer drug (DREADD) hM4Di prevented attractive behavior to chemosignals of the opposite sex (DiBenedictis et al., 2015). Notably, chemogenetic silencing of the medial OT did not suppress attractive behaviors to peanut butter odor in pre-fed mice, implying that the medial OT is dispensable and is not the sole neural circuit that facilitates attraction to food-related odors. The medial OT receives dopaminergic inputs from the medial part of the ventral tegmental area (VTA) (Ikemoto, 2007). Optogenetic stimulation of dopaminergic axon terminals of the VTA-medial OT pathway promoted place preference and odor preference (Zhang et al., 2017a). Neuronal subtype-specific activation of D1 or D2 receptor-expressing neurons has provided further insight into the functions of the anteromedial OT. Optogenetic stimulation of D1 receptor-expressing neurons and D2 receptor-expressing neurons in the anteromedial OT elicited place preference and place aversion, respectively (Murata et al., 2019b). The effects of manipulating the lateral domain of the OT on aversive behaviors are yet to be reported. Neuroanatomical tracing has revealed that the cap regions in the lateral OT receive axonal projections from tufted cells in the dorsal part of the olfactory bulb, which respond to predator fox odor trimethylthiazoline and rotten food odor 2-methylbutyric acid (Igarashi et al., 2012). Further experiments are required to elucidate the spatially segregated domain- and neuronal subtype-specific functional roles of the OT in odor-guided motivated behaviors.

### Involvement of the Olfactory Tubercle and Nucleus Accumbens in Motivated and Hedonic Eating

Another component of the ventral striatum, the NAc, constitutes a critical node within mesocorticolimbic circuits that mediate “wanting” and “liking” (Richard et al., 2013; Morales and Berridge, 2020). Pharmacological functional mapping revealed that local microcircuits of the NAc medial shell differentially regulate food intake of palatable chocolate and hedonic reactions to taste stimulation. Microinjections of the GABA_A_ receptor agonist muscimol into the rostral medial shell elicited increased food intake and hedonic reactions to the taste of sucrose. In contrast, microinjections of muscimol into the caudal medial shell induced defensive behavior and aversive reactions to sucrose or quinine tastants (Reynolds and Berridge, 2002). Microinjections of agonists of opioid receptor subtypes (mu, delta, and kappa) have revealed the anatomical heterogeneity of the NAc in the regulation of motivated and hedonic eating behaviors (Castro and Berridge, 2014). The anatomical similarity between the OT andNAc, both of which contain GABAergic neurons that project to the ventral pallidum, raises the possibility of similar functional maps of motivated and hedonic behaviors in the OT (Zahm and Heimer, 1985; Heimer et al., 1987; Zhou et al., 2003). Optogenetic self-stimulation experiments suggested that the excitation of D1 receptor-expressing neurons in the medial shell of the NAc supports strong incentive motivation to self-stimulate (Cole et al., 2018). However, neural pathways from the OT are not identical to those from the NAc. For instance, D1 receptor-expressing medium spiny neurons in the NAc provide synaptic inputs onto GABAergic neurons in the lateral hypothalamus, which are involved in the downregulation of feeding behavior (O’Connor et al., 2015). Retrograde tracing from the lateral hypothalamus revealed a substantially larger number of labeled cells in the NAc than in the OT (Murata et al., 2019a). Future studies should address whether and how distinct OT domains regulate motivated and hedonic eating behaviors.

The development of OT neural circuits during weaning has implications for the involvement of the OT in eating. Neurogenesis of the OT in the embryonic mouse and rat occurs in a lateral-to-medial gradient (Bayer, 1985; Martin-Lopez et al., 2019). Early postnatal development of the mouse OT is consistent with this gradient (Martin-Lopez et al., 2019). *Drd1* mRNA and DARPP-32 expression in the lateral OT precedes the corresponding expression that in the anteromedial OT at postnatal days 3-8 (P3-8) (Murofushi et al., 2018). Mapping of c-fos expression after search and consumption of food pellets indicated widespread neural activation across diffuse OT domains at P15, whereas the anteromedial domain was preferentially activated at P21 and later ages (Murofushi et al., 2018). Acquisition of food-eating habits including consumption of laboratory animal pellets is a form of learning during weaning which involves evaluation, motivation, and palatability of foods, which may be associated with the functional maturation of OT domains.

The OT receives multimodal sensory inputs alongside olfaction (Wesson and Wilson, 2011). Odor information can be conveyed to the OT directly from the olfactory bulb or via other areas of the olfactory cortex (Heimer, 1978). Whole-brain mapping of input pathways has indicated that the medial OT receives synaptic inputs from widespread areas of the brain including the isocortex, septum, striatum, pallidum, amygdala, thalamus, hypothalamus, olfactory areas, and VTA (Zhang et al., 2017b). The VTA-medial OT dopaminergic pathway is activated by rewarding experiences such as sucrose-licking (Zhang et al., 2017a). Axonal projections from the nucleus of the solitary tract to the OT suggest that visceral signals are directly transferred to the OT via this pathway (Ruggiero et al., 1998). The OT expresses various receptors of neuromodulatory inputs, opioids, hormones, and neurosteroids (Cansler et al., 2020). In the anteromedial OT, various orexigenic peptides (orexin and prodynorphin) and receptors (orexin receptors 1 and 2, ghrelin receptor, and opioid receptor kappa 1) were highly expressed compared to those in the lateral OT. This trend was also seen in anorexigenic peptides (CART peptide) and receptors (melanocortin four receptor and arginine-vasopressin receptor 1a) (Nogi et al., 2020). Odor cues may influence the release and expression of these feeding-related peptides and receptors in the anteromedial OT and the subsequent ingestive behaviors. Indeed, the expression levels of the orexigenic peptide (ghrelin) and receptors (cannabinoid receptor 1, opioid receptor delta 1, and opioid receptor kappa 1) were increased in the anteromedial OT by odor-guided food-seeking behavior. A similar increase was noted in the levels of the anorexigenic peptide (arginine-vasopressin) and receptors (leptin receptor and melanocortin four receptor) in the anteromedial OT (Nogi et al., 2020). Cell-type identification will assist in determining how specific neural circuits in the OT function in eating behaviors by elucidating which neurons (D1 receptor or D2 receptor-expressing neurons and other types of neurons) express the aforementioned peptides and receptors.

### A Hypothetical Neural Model Relating the OT and Eating Behavior

In summary, the OT has the following neuroanatomical and neurochemical similarities with the NAc: dense dopaminergic inputs from the VTA, high expression of the dopamine receptors, and GABAergic outputs to the ventral pallidum (Heimer et al., 1987; Ikemoto, 2007). A notable difference between the OT and NAc is that the OT receives abundant synaptic inputs from the olfactory bulb and olfactory cortical areas (Zhang et al., 2017b), which convey olfactory sensory cues of food in the environment and olfactory components of flavors during mastication via the retronasal pathway to the OT. Indeed, the OT is considered the “striatum with olfactory function” (Wesson and Wilson, 2011; Martin-Lopez et al., 2019) and may drive olfaction-based “wanting” and “liking” behaviors (Richard et al., 2013; Morales and Berridge, 2020).

**FIGURE 2:**
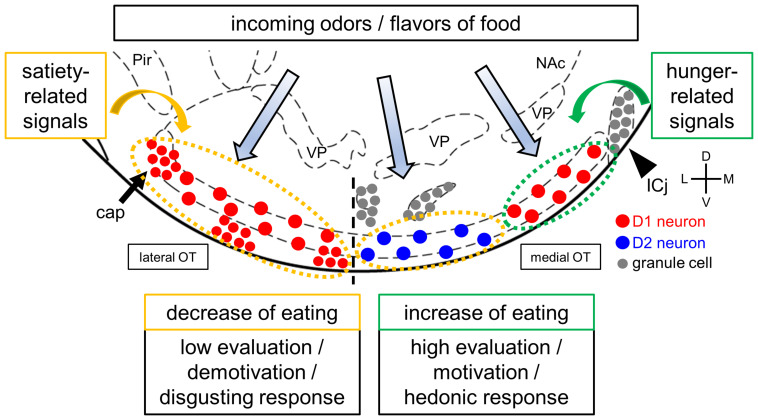
A hypothetical model of the OT and eating. The OT receives and may integrate olfactory inputs and feeding-related hormonal signals, as well as other sensory modalities, visceral, and neuromodulatory inputs. The hypothesis is that activation of the D1 receptor-expressing neurons in the anteromedial OT increases food intake, whereas activation of the D2 receptor-expressing neurons in the anteromedial OT and D1 receptor-expressing neurons in the lateral OT decreases food intake based on their involvement in attractive and aversive behaviors, respectively. Feeding-related hormonal signals, such hunger and satiety, may regulate the activity of OT neurons in a domain- and cell type-specific manner. These signals result in homeostatic evaluation, motivation, and hedonic responses to food odors and flavors. D1 and D2 receptor-expressing neurons in layer II of the cortex-like region were noted to be intermingled in both the anteromedial and lateral OT domains, as shown in Figure 1 but simplified here in this figure. Red circles, D1 receptor-expressing medium spiny neurons in layer II of cortex-like regions and dwarf cells in the cap regions (arrows); blue circles, D2 receptor-expressing medium spiny neurons in layer II of cortex-like regions; gray circles, granule cells in the Islands of Calleja (arrowheads). Pir, piriform cortex; NAc, nucleus accumbens; VP, ventral pallidum; ICj, Islands of Calleja. D, dorsal; V, ventral; M, medial; L, lateral. Stereotaxic atlas from Paxinos and Franklin (2008).

As previously mentioned, the cytoarchitectonically defined-domains of the mouse OT play distinct roles in attractive and aversive responses to odor cues (Figure 1). These domains raise the possibility that D1 receptor-expressing neurons in the anteromedial domain facilitate eating by high evaluation, motivation, and hedonic response to food-related odors and flavors. In contrast, D2 receptor-expressing neurons in the anteromedial domain and D1 receptor-expressing neurons in the lateral domain may suppress eating by low evaluation, demotivation, and disgusting response to food-related odors and flavors. The OT also expresses orexigenic and anorexigenic peptides and their receptors (Cansler et al., 2020; Nogi et al., 2020), which may endow OT domains with homeostatic control over odor-guided eating behaviors (Aime et al., 2007; Palouzier-Paulignan et al., 2012). For instance, hunger state and its orexigenic hormonal signals such as orexin and ghrelin might upregulate the responses of D1 receptor-expressing neurons in the anteromedial OT to food odors and flavors, resulting in increase of food intake. Satiety state and its anorexigenic hormonal signals such as leptin, in contrast, might upregulate responses to food odors and flavors of D2 receptor-expressing neurons in the anteromedial OT and D1 receptor-expressing neurons in the lateral OT, resulting in food intake suppression (Figure 2). Future studies should address whether and how the OT integrates olfaction, other sensory modalities, and visceral and feeding-related endocrine signals during and after eating. Specific domains and neuronal subtypes of the OT may be activated by incoming environmental odors and flavors of food when evaluating the decision to eat, appetitive or aversive motivation, and hedonic palatability.

## Author Contributions

KM wrote the manuscript.

## Conflict of Interest

The author declares that the research was conducted in the absence of any commercial or financial relationships that could be construed as a potential conflict of interest.
